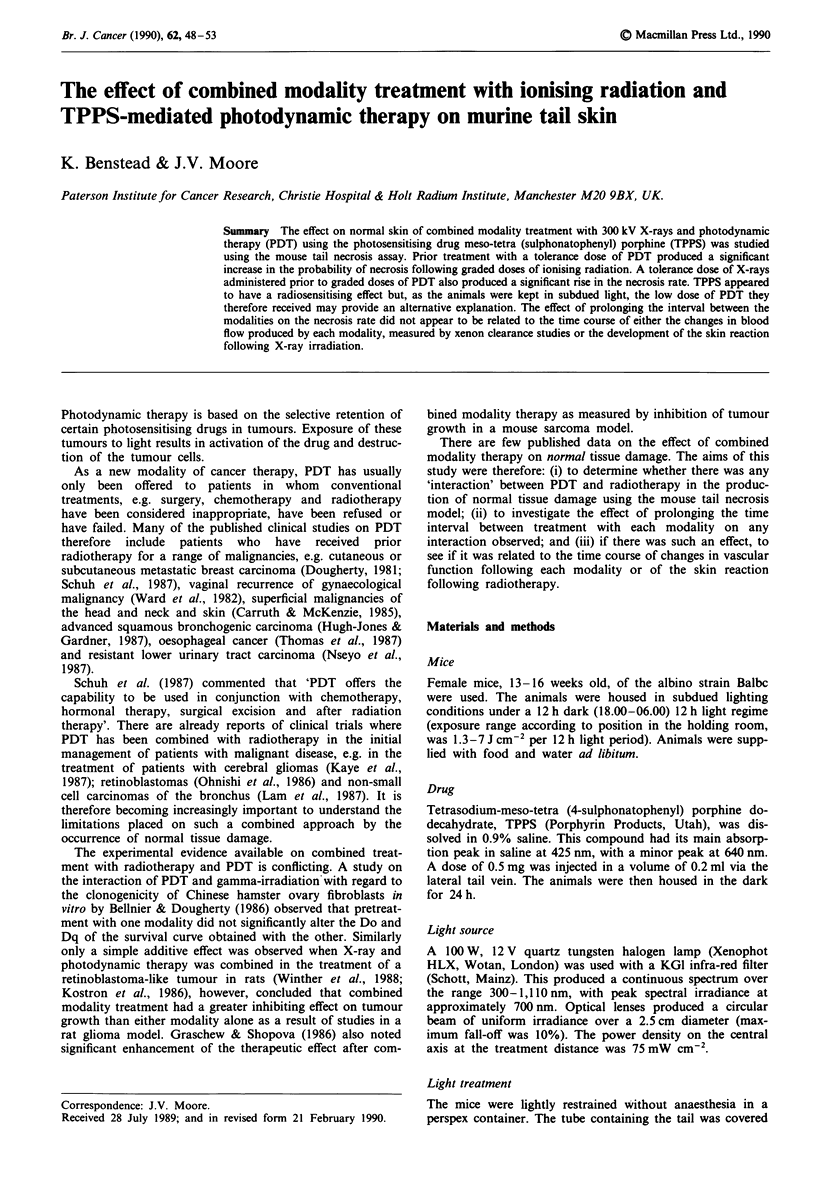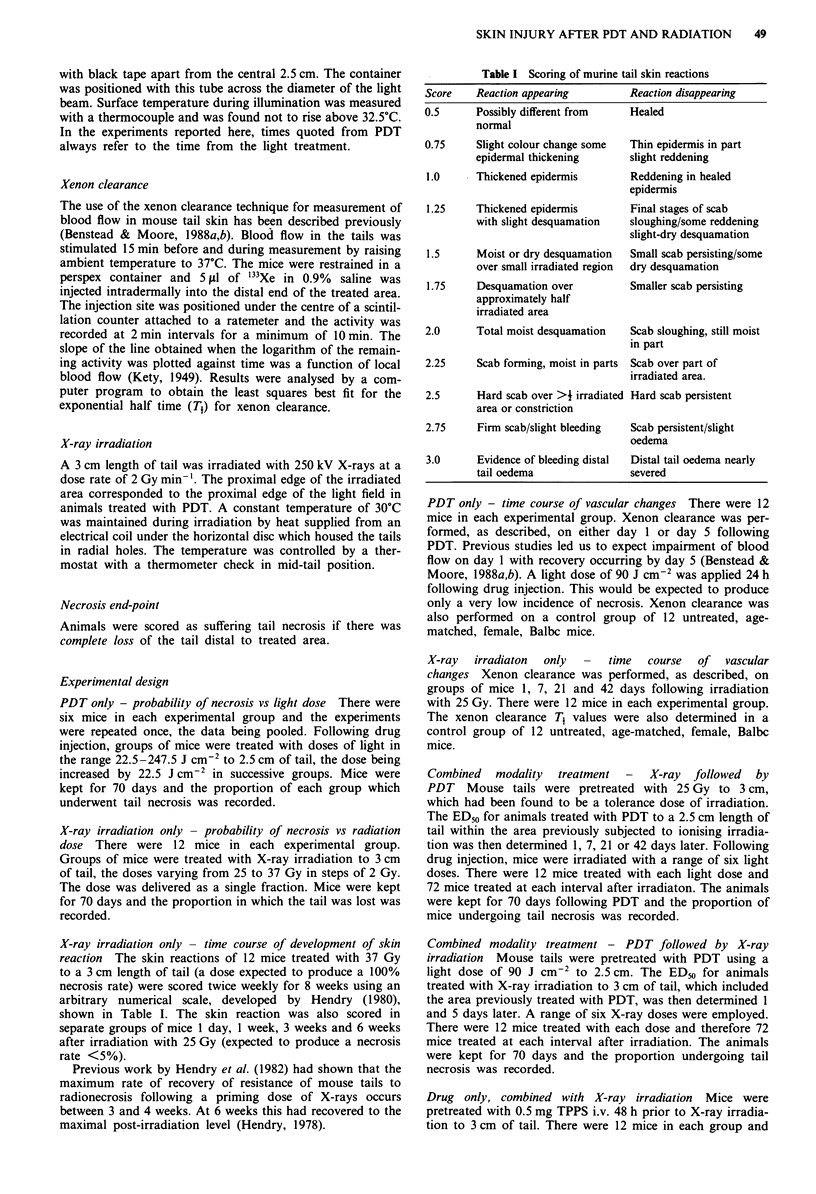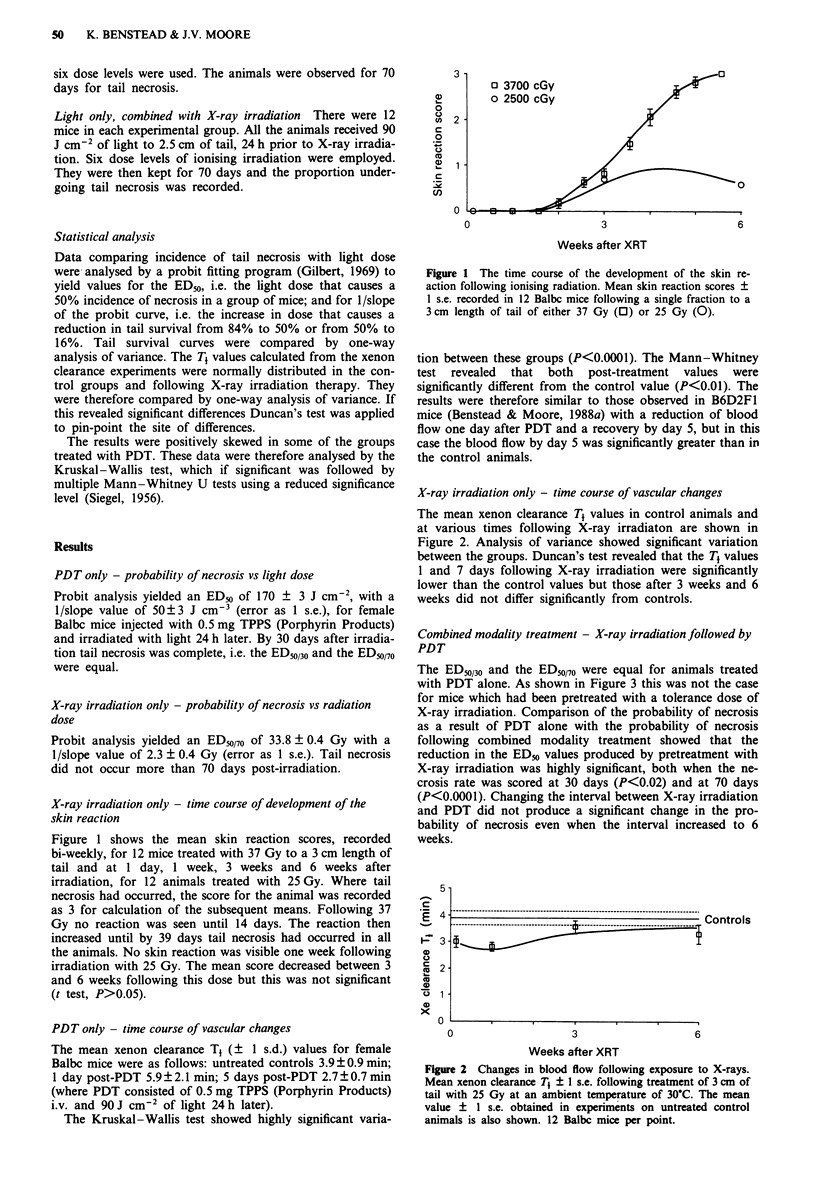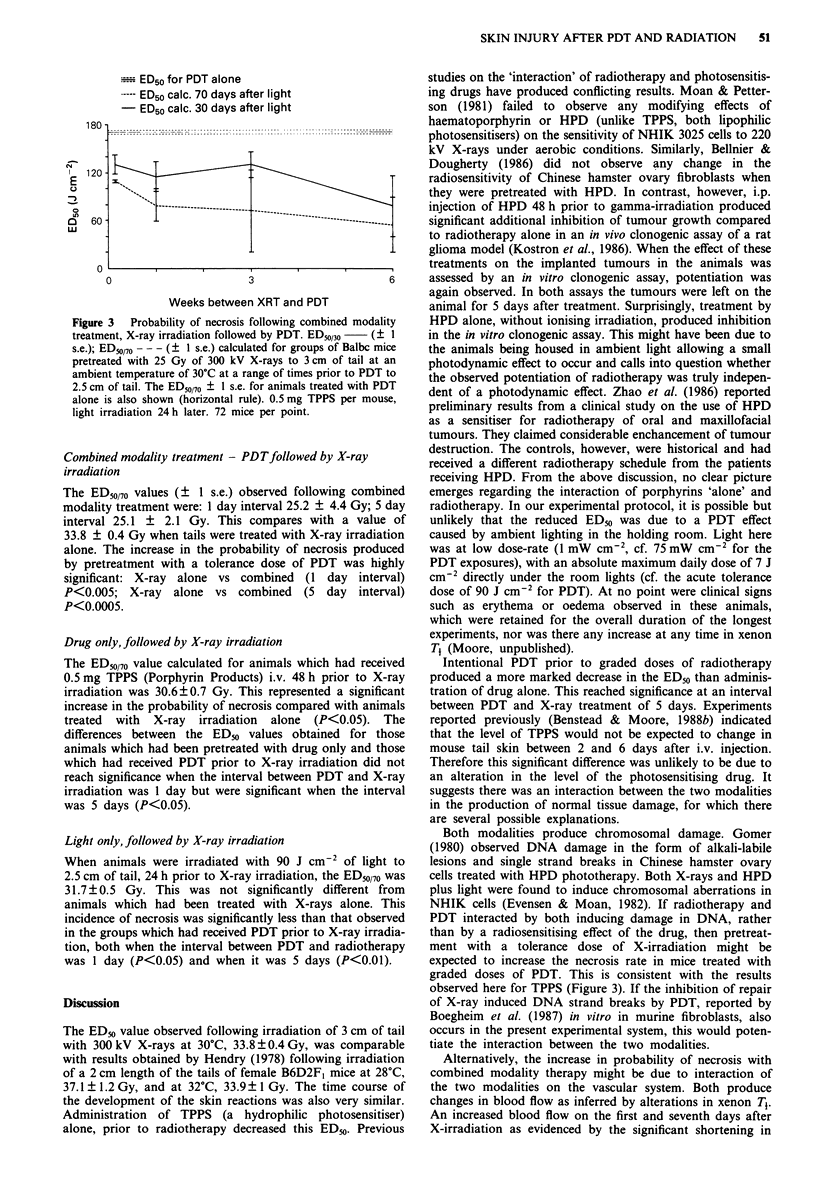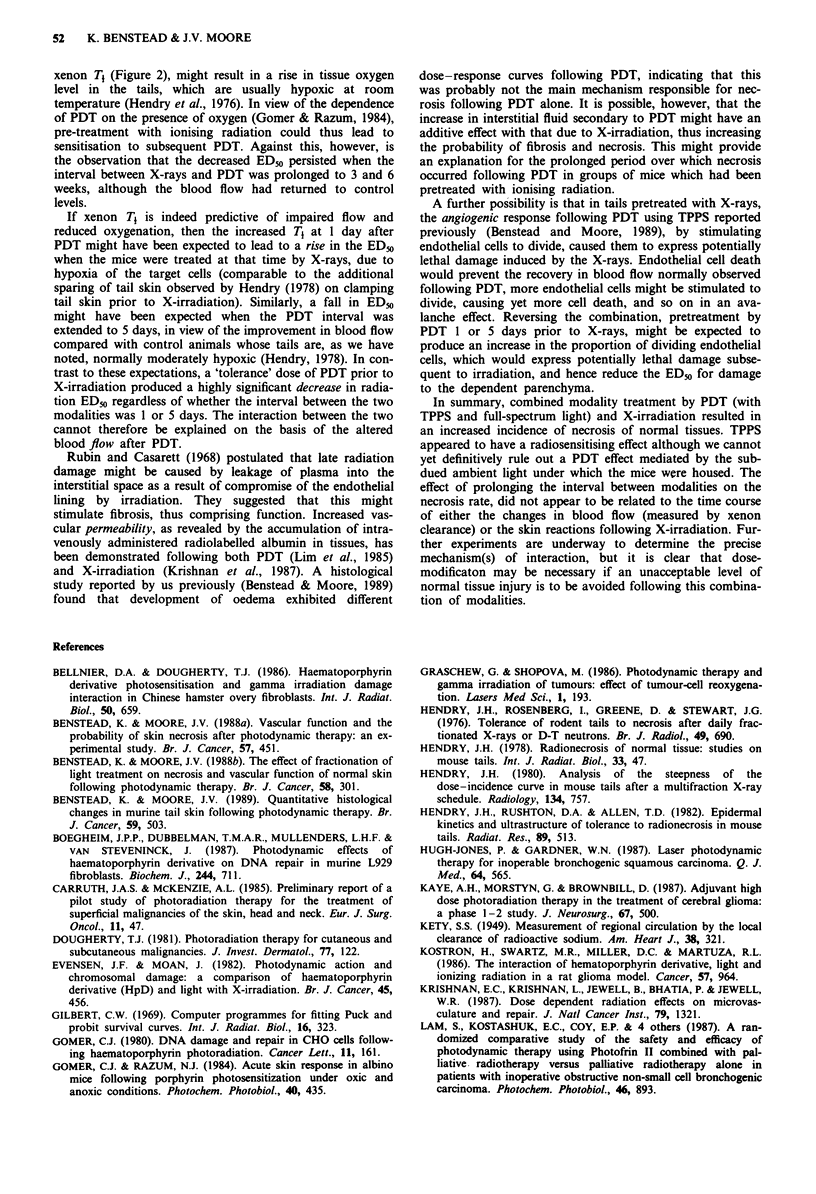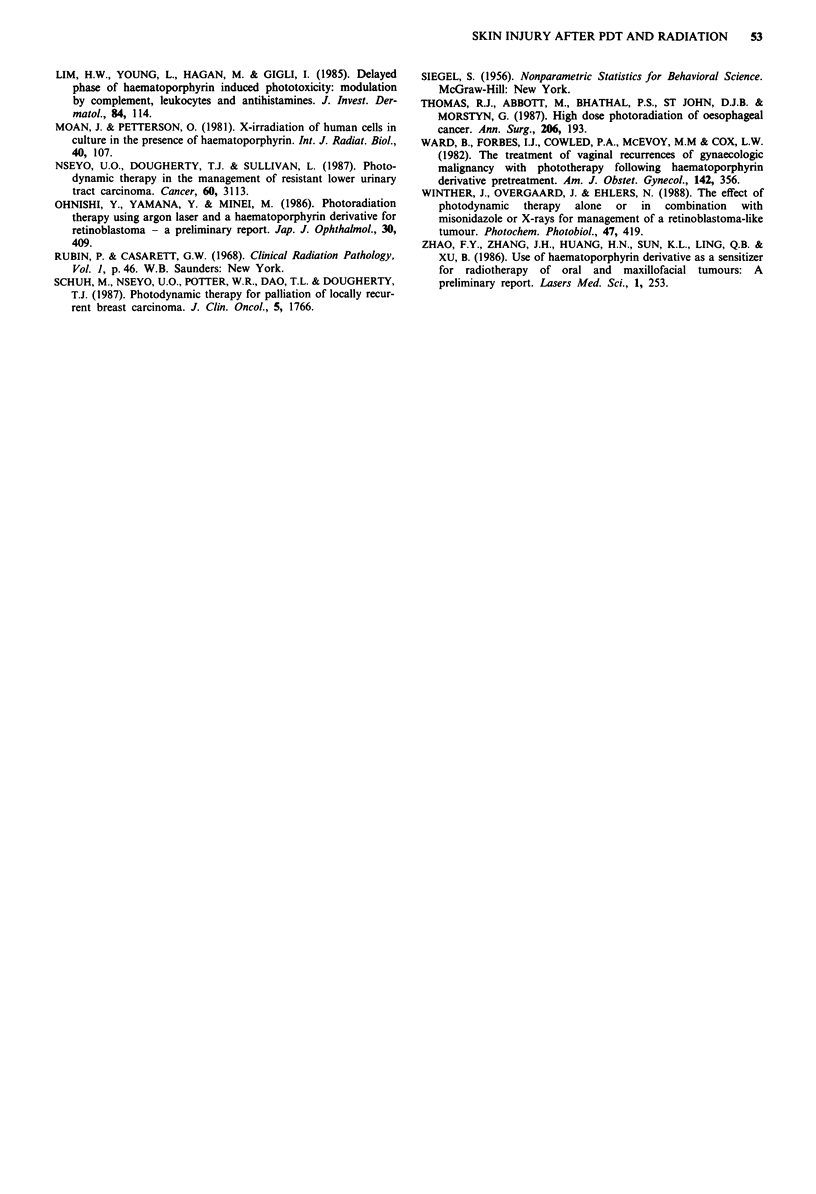# The effect of combined modality treatment with ionising radiation and TPPS-mediated photodynamic therapy on murine tail skin.

**DOI:** 10.1038/bjc.1990.227

**Published:** 1990-07

**Authors:** K. Benstead, J. V. Moore

**Affiliations:** Paterson Institute for Cancer Research, Christie Hospital, Manchester, UK.

## Abstract

The effect on normal skin of combined modality treatment with 300 kV X-rays and photodynamic therapy (PDT) using the photosensitising drug meso-tetra (sulphonatophenyl) porphine (TPPS) was studied using the mouse tail necrosis assay. Prior treatment with a tolerance dose of PDT produced a significant increase in the probability of necrosis following graded doses of ionising radiation. A tolerance dose of X-rays administered prior to graded doses of PDT also produced a significant rise in the necrosis rate. TPPS appeared to have a radiosensitising effect but, as the animals were kept in subdued light, the low dose of PDT they therefore received may provide an alternative explanation. The effect of prolonging the interval between the modalities on the necrosis rate did not appear to be related to the time course of either the changes in blood flow produced by each modality, measured by xenon clearance studies or the development of the skin reaction following X-ray irradiation.


Br. J. Cancer (1990), 62, 48-53 © Macmillan Press Ltd., 1990

### The effect of combined modality treatment with ionising radiation and TPPS-mediated photodynamic therapy on murine tail skin

K. Benstead & J.V. Moore

*Paterson Institute for Cancer Research, Christie Hospital & Holt Radium Institute, Manchester M20 9BX, UK.*

**Summary** The effect on normal skin of combined modality treatment with 300 kV X-rays and photodynamic therapy (PDT) using the photosensitising drug meso-tetra (sulphonatophenyl) porphine (TPPS) was studied using the mouse tail necrosis assay. Prior treatment with a tolerance dose of PDT produced a significant increase in the probability of necrosis following graded doses of ionising radiation. A tolerance dose of X-rays administered prior to graded doses of PDT also produced a significant rise in the necrosis rate. TPPS appeared to have a radiosensitising effect but, as the animals were kept in subdued light, the low dose of PDT they therefore received may provide an alternative explanation. The effect of prolonging the interval between the modalities on the necrosis rate did not appear to be related to the time course of either the changes in blood flow produced by each modality, measured by xenon clearance studies or the development of the skin reaction following X-ray irradiation.

Photodynamic therapy is based on the selective retention of certain photosensitising drugs in tumours. Exposure of these tumours to light results in activation of the drug and destruction of the tumour cells.

As a new modality of cancer therapy, PDT has usually only been offered to patients in whom conventional treatments, e.g. surgery, chemotherapy and radiotherapy have been considered inappropriate, have been refused or have failed. Many of the published clinical studies on PDT therefore include patients who have received prior radiotherapy for a range of malignancies, e.g. cutaneous or subcutaneous metastatic breast carcinoma (Dougherty, 1981; Schuh *et al.*, 1987), vaginal recurrence of gynaecological malignancy (Ward *et al.*, 1982), superficial malignancies of the head and neck and skin (Carruth & McKenzie, 1985), advanced squamous bronchogenic carcinoma (Hugh-Jones & Gardner, 1987), oesophageal cancer (Thomas *et al.*, 1987) and resistant lower urinary tract carcinoma (Nseyo *et al.*, 1987).

Schuh *et al.* (1987) commented that 'PDT offers the capability to be used in conjunction with chemotherapy, hormonal therapy, surgical excision and after radiation therapy'. There are already reports of clinical trials where PDT has been combined with radiotherapy in the initial management of patients with malignant disease, e.g. in the treatment of patients with cerebral gliomas (Kaye *et al.*, 1987); retinoblastomas (Ohnishi *et al.*, 1986) and non-small cell carcinomas of the bronchus (Lam *et al.*, 1987). It is therefore becoming increasingly important to understand the limitations placed on such a combined approach by the occurrence of normal tissue damage.

The experimental evidence available on combined treatment with radiotherapy and PDT is conflicting. A study on the interaction of PDT and gamma-irradiation with regard to the clonogenicity of Chinese hamster ovary fibroblasts *in vitro* by Bellnier & Dougherty (1986) observed that pretreatment with one modality did not significantly alter the Do and Dq of the survival curve obtained with the other. Similarly only a simple additive effect was observed when X-ray and photodynamic therapy was combined in the treatment of a retinoblastoma-like tumour in rats (Winther *et al.*, 1988; Kostron *et al.*, 1986), however, concluded that combined modality treatment had a greater inhibiting effect on tumour growth than either modality alone as a result of studies in a rat glioma model. Graschew & Shopova (1986) also noted significant enhancement of the therapeutic effect after combined modality therapy as measured by inhibition of tumour growth in a mouse sarcoma model.

There are few published data on the effect of combined modality therapy on *normal* tissue damage. The aims of this study were therefore: (i) to determine whether there was any 'interaction' between PDT and radiotherapy in the production of normal tissue damage using the mouse tail necrosis model; (ii) to investigate the effect of prolonging the time interval between treatment with each modality on any interaction observed; and (iii) if there was such an effect, to see if it was related to the time course of changes in vascular function following each modality or of the skin reaction following radiotherapy.

### Materials and methods

### *Mice*

Female mice, 13-16 weeks old, of the albino strain Balbc were used. The animals were housed in subdued lighting conditions under a 12 h dark (18.00-06.00) 12 h light regime (exposure range according to position in the holding room, was 1.3-7 J $cm^{-2}$ per 12 h light period). Animals were supplied with food and water *ad libitum*.

### *Drug*

Tetrasodium-meso-tetra (4-sulphonatophenyl) porphine dodecahydrate, TPPS (Porphyrin Products, Utah), was dissolved in 0.9% saline. This compound had its main absorption peak in saline at 425 nm, with a minor peak at 640 nm. A dose of 0.5 mg was injected in a volume of 0.2 ml via the lateral tail vein. The animals were then housed in the dark for 24 h.

### *Light source*

A 100 W, 12 V quartz tungsten halogen lamp (Xenophot HLX, Wotan, London) was used with a KGl infra-red filter (Schott, Mainz). This produced a continuous spectrum over the range 300-1,110 nm, with peak spectral irradiance at approximately 700 nm. Optical lenses produced a circular beam of uniform irradiance over a 2.5 cm diameter (maximum fall-off was 10%). The power density on the central axis at the treatment distance was 75 mW $cm^{-2}$.

### *Light treatment*

The mice were lightly restrained without anaesthesia in a perspex container. The tube containing the tail was covered

Correspondence: J.V. Moore.
Received 28 July 1989; and in revised form 21 February 1990.

with black tape apart from the central 2.5 cm. The container was positioned with this tube across the diameter of the light beam. Surface temperature during illumination was measured with a thermocouple and was found not to rise above 32.5°C. In the experiments reported here, times quoted from PDT always refer to the time from the light treatment.

### *Xenon clearance*

The use of the xenon clearance technique for measurement of blood flow in mouse tail skin has been described previously (Benstead & Moore, 1988*a*,*b*). Blood flow in the tails was stimulated 15 min before and during measurement by raising ambient temperature to 37°C. The mice were restrained in a perspex container and 5 µl of $^{133}Xe$ in 0.9% saline was injected intradermally into the distal end of the treated area. The injection site was positioned under the centre of a scintillation counter attached to a ratemeter and the activity was recorded at 2 min intervals for a minimum of 10 min. The slope of the line obtained when the logarithm of the remaining activity was plotted against time was a function of local blood flow (Kety, 1949). Results were analysed by a computer program to obtain the least squares best fit for the exponential half time ($T_{\frac{1}{2}}$) for xenon clearance.

### *X-ray irradiation*

A 3 cm length of tail was irradiated with 250 kV X-rays at a dose rate of 2 Gy min$^{-1}$. The proximal edge of the irradiated area corresponded to the proximal edge of the light field in animals treated with PDT. A constant temperature of 30°C was maintained during irradiation by heat supplied from an electrical coil under the horizontal disc which housed the tails in radial holes. The temperature was controlled by a thermostat with a thermometer check in mid-tail position.

### *Necrosis end-point*

Animals were scored as suffering tail necrosis if there was *complete loss* of the tail distal to treated area.

### *Experimental design*

*PDT only – probability of necrosis vs light dose* There were six mice in each experimental group and the experiments were repeated once, the data being pooled. Following drug injection, groups of mice were treated with doses of light in the range 22.5–247.5 J cm$^{-2}$ to 2.5 cm of tail, the dose being increased by 22.5 J cm$^{-2}$ in successive groups. Mice were kept for 70 days and the proportion of each group which underwent tail necrosis was recorded.

*X-ray irradiation only – probability of necrosis vs radiation dose* There were 12 mice in each experimental group. Groups of mice were treated with X-ray irradiation to 3 cm of tail, the doses varying from 25 to 37 Gy in steps of 2 Gy. The dose was delivered as a single fraction. Mice were kept for 70 days and the proportion in which the tail was lost was recorded.

*X-ray irradiation only – time course of development of skin reaction* The skin reactions of 12 mice treated with 37 Gy to a 3 cm length of tail (a dose expected to produce a 100% necrosis rate) were scored twice weekly for 8 weeks using an arbitrary numerical scale, developed by Hendry (1980), shown in Table I. The skin reaction was also scored in separate groups of mice 1 day, 1 week, 3 weeks and 6 weeks after irradiation with 25 Gy (expected to produce a necrosis rate <5%).

Previous work by Hendry *et al.* (1982) had shown that the maximum rate of recovery of resistance of mouse tails to radionecrosis following a priming dose of X-rays occurs between 3 and 4 weeks. At 6 weeks this had recovered to the maximal post-irradiation level (Hendry, 1978).

**Table I** Scoring of murine tail skin reactions

| *Score* | *Reaction appearing* | *Reaction disappearing* |
|---|---|---|
| 0.5 | Possibly different from normal | Healed |
| 0.75 | Slight colour change some epidermal thickening | Thin epidermis in part slight reddening |
| 1.0 | Thickened epidermis | Reddening in healed epidermis |
| 1.25 | Thickened epidermis with slight desquamation | Final stages of scab sloughing/some reddening slight-dry desquamation |
| 1.5 | Moist or dry desquamation over small irradiated region | Small scab persisting/some dry desquamation |
| 1.75 | Desquamation over approximately half irradiated area | Smaller scab persisting |
| 2.0 | Total moist desquamation | Scab sloughing, still moist in part |
| 2.25 | Scab forming, moist in parts | Scab over part of irradiated area. |
| 2.5 | Hard scab over >½ irradiated area or constriction | Hard scab persistent |
| 2.75 | Firm scab/slight bleeding | Scab persistent/slight oedema |
| 3.0 | Evidence of bleeding distal tail oedema | Distal tail oedema nearly severed |

*PDT only – time course of vascular changes* There were 12 mice in each experimental group. Xenon clearance was performed, as described, on either day 1 or day 5 following PDT. Previous studies led us to expect impairment of blood flow on day 1 with recovery occurring by day 5 (Benstead & Moore, 1988*a*,*b*). A light dose of 90 J cm$^{-2}$ was applied 24 h following drug injection. This would be expected to produce only a very low incidence of necrosis. Xenon clearance was also performed on a control group of 12 untreated, age-matched, female, Balbc mice.

*X-ray irradiaton only – time course of vascular changes* Xenon clearance was performed, as described, on groups of mice 1, 7, 21 and 42 days following irradiation with 25 Gy. There were 12 mice in each experimental group. The xenon clearance $T_{\frac{1}{2}}$ values were also determined in a control group of 12 untreated, age-matched, female, Balbc mice.

*Combined modality treatment – X-ray followed by PDT* Mouse tails were pretreated with 25 Gy to 3 cm, which had been found to be a tolerance dose of irradiation. The $ED_{50}$ for animals treated with PDT to a 2.5 cm length of tail within the area previously subjected to ionising irradiation was then determined 1, 7, 21 or 42 days later. Following drug injection, mice were irradiated with a range of six light doses. There were 12 mice treated with each light dose and 72 mice treated at each interval after irradiaton. The animals were kept for 70 days following PDT and the proportion of mice undergoing tail necrosis was recorded.

*Combined modality treatment – PDT followed by X-ray irradiation* Mouse tails were pretreated with PDT using a light dose of 90 J cm$^{-2}$ to 2.5 cm. The $ED_{50}$ for animals treated with X-ray irradiation to 3 cm of tail, which included the area previously treated with PDT, was then determined 1 and 5 days later. A range of six X-ray doses were employed. There were 12 mice treated with each dose and therefore 72 mice treated at each interval after irradiation. The animals were kept for 70 days and the proportion undergoing tail necrosis was recorded.

*Drug only, combined with X-ray irradiation* Mice were pretreated with 0.5 mg TPPS i.v. 48 h prior to X-ray irradiation to 3 cm of tail. There were 12 mice in each group and six dose levels were used. The animals were observed for 70 days for tail necrosis.

*Light only, combined with X-ray irradiation* There were 12 mice in each experimental group. All the animals received 90 J $cm^{-2}$ of light to 2.5 cm of tail, 24 h prior to X-ray irradiation. Six dose levels of ionising irradiation were employed. They were then kept for 70 days and the proportion undergoing tail necrosis was recorded.

*Statistical analysis*

Data comparing incidence of tail necrosis with light dose were analysed by a probit fitting program (Gilbert, 1969) to yield values for the $ED_{50}$, i.e. the light dose that causes a 50% incidence of necrosis in a group of mice; and for 1/slope of the probit curve, i.e. the increase in dose that causes a reduction in tail survival from 84% to 50% or from 50% to 16%. Tail survival curves were compared by one-way analysis of variance. The $T_{\frac{1}{2}}$ values calculated from the xenon clearance experiments were normally distributed in the control groups and following X-ray irradiation therapy. They were therefore compared by one-way analysis of variance. If this revealed significant differences Duncan's test was applied to pin-point the site of differences.

The results were positively skewed in some of the groups treated with PDT. These data were therefore analysed by the Kruskal-Wallis test, which if significant was followed by multiple Mann-Whitney U tests using a reduced significance level (Siegel, 1956).

### Results

*PDT only – probability of necrosis vs light dose*

Probit analysis yielded an $ED_{50}$ of 170 ± 3 J $cm^{-2}$, with a 1/slope value of 50±3 J $cm^{-3}$ (error as 1 s.e.), for female Balbc mice injected with 0.5 mg TPPS (Porphyrin Products) and irradiated with light 24 h later. By 30 days after irradiation tail necrosis was complete, i.e. the $ED_{50/30}$ and the $ED_{50/70}$ were equal.

*X-ray irradiation only – probability of necrosis vs radiation dose*

Probit analysis yielded an $ED_{50/70}$ of 33.8 ± 0.4 Gy with a 1/slope value of 2.3 ± 0.4 Gy (error as 1 s.e.). Tail necrosis did not occur more than 70 days post-irradiation.

*X-ray irradiation only – time course of development of the skin reaction*

Figure 1 shows the mean skin reaction scores, recorded bi-weekly, for 12 mice treated with 37 Gy to a 3 cm length of tail and at 1 day, 1 week, 3 weeks and 6 weeks after irradiation, for 12 animals treated with 25 Gy. Where tail necrosis had occurred, the score for the animal was recorded as 3 for calculation of the subsequent means. Following 37 Gy no reaction was seen until 14 days. The reaction then increased until by 39 days tail necrosis had occurred in all the animals. No skin reaction was visible one week following irradiation with 25 Gy. The mean score decreased between 3 and 6 weeks following this dose but this was not significant (*t* test, $P>0.05$).

*PDT only – time course of vascular changes*

The mean xenon clearance $T_{\frac{1}{2}}$ (± 1 s.d.) values for female Balbc mice were as follows: untreated controls 3.9±0.9 min; 1 day post-PDT 5.9±2.1 min; 5 days post-PDT 2.7±0.7 min (where PDT consisted of 0.5 mg TPPS (Porphyrin Products) i.v. and 90 J $cm^{-2}$ of light 24 h later).

The Kruskal-Wallis test showed highly significant variation between these groups ($P<0.0001$). The Mann-Whitney test revealed that both post-treatment values were significantly different from the control value ($P<0.01$). The results were therefore similar to those observed in B6D2F1 mice (Benstead & Moore, 1988*a*) with a reduction of blood flow one day after PDT and a recovery by day 5, but in this case the blood flow by day 5 was significantly greater than in the control animals.

**Figure 1** The time course of the development of the skin reaction following ionising radiation. Mean skin reaction scores ± 1 s.e. recorded in 12 Balbc mice following a single fraction to a 3 cm length of tail of either 37 Gy (□) or 25 Gy (○).

*X-ray irradiation only – time course of vascular changes*

The mean xenon clearance $T_{\frac{1}{2}}$ values in control animals and at various times following X-ray irradiaton are shown in Figure 2. Analysis of variance showed significant variation between the groups. Duncan's test revealed that the $T_{\frac{1}{2}}$ values 1 and 7 days following X-ray irradiation were significantly lower than the control values but those after 3 weeks and 6 weeks did not differ significantly from controls.

*Combined modality treatment – X-ray irradiation followed by PDT*

The $ED_{50/30}$ and the $ED_{50/70}$ were equal for animals treated with PDT alone. As shown in Figure 3 this was not the case for mice which had been pretreated with a tolerance dose of X-ray irradiation. Comparison of the probability of necrosis as a result of PDT alone with the probability of necrosis following combined modality treatment showed that the reduction in the $ED_{50}$ values produced by pretreatment with X-ray irradiation was highly significant, both when the necrosis rate was scored at 30 days ($P<0.02$) and at 70 days ($P<0.0001$). Changing the interval between X-ray irradiation and PDT did not produce a significant change in the probability of necrosis even when the interval increased to 6 weeks.

**Figure 2** Changes in blood flow following exposure to X-rays. Mean xenon clearance $T_{\frac{1}{2}}$ ± 1 s.e. following treatment of 3 cm of tail with 25 Gy at an ambient temperature of 30°C. The mean value ± 1 s.e. obtained in experiments on untreated control animals is also shown. 12 Balbc mice per point.

**Figure 3** Probability of necrosis following combined modality treatment, X-ray irradiation followed by PDT. $ED_{50/30}$ —— (± 1 s.e.); $ED_{50/70}$ - - - (± 1 s.e.) calculated for groups of Balbc mice pretreated with 25 Gy of 300 kV X-rays to 3 cm of tail at an ambient temperature of 30°C at a range of times prior to PDT to 2.5 cm of tail. The $ED_{50/70}$ ± 1 s.e. for animals treated with PDT alone is also shown (horizontal rule). 0.5 mg TPPS per mouse, light irradiation 24 h later. 72 mice per point.

*Combined modality treatment – PDT followed by X-ray irradiation*

The $ED_{50/70}$ values (± 1 s.e.) observed following combined modality treatment were: 1 day interval 25.2 ± 4.4 Gy; 5 day interval 25.1 ± 2.1 Gy. This compares with a value of 33.8 ± 0.4 Gy when tails were treated with X-ray irradiation alone. The increase in the probability of necrosis produced by pretreatment with a tolerance dose of PDT was highly significant: X-ray alone vs combined (1 day interval) $P<0.005$; X-ray alone vs combined (5 day interval) $P<0.0005$.

*Drug only, followed by X-ray irradiation*

The $ED_{50/70}$ value calculated for animals which had received 0.5 mg TPPS (Porphyrin Products) i.v. 48 h prior to X-ray irradiation was 30.6±0.7 Gy. This represented a significant increase in the probability of necrosis compared with animals treated with X-ray irradiation alone ($P<0.05$). The differences between the $ED_{50}$ values obtained for those animals which had been pretreated with drug only and those which had received PDT prior to X-ray irradiation did not reach significance when the interval between PDT and X-ray irradiation was 1 day but were significant when the interval was 5 days ($P<0.05$).

*Light only, followed by X-ray irradiation*

When animals were irradiated with 90 J $cm^{-2}$ of light to 2.5 cm of tail, 24 h prior to X-ray irradiation, the $ED_{50/70}$ was 31.7±0.5 Gy. This was not significantly different from animals which had been treated with X-rays alone. This incidence of necrosis was significantly less than that observed in the groups which had received PDT prior to X-ray irradiation, both when the interval between PDT and radiotherapy was 1 day ($P<0.05$) and when it was 5 days ($P<0.01$).

### Discussion

The $ED_{50}$ value observed following irradiation of 3 cm of tail with 300 kV X-rays at 30°C, 33.8±0.4 Gy, was comparable with results obtained by Hendry (1978) following irradiation of a 2 cm length of the tails of female $B6D2F_1$ mice at 28°C, 37.1±1.2 Gy, and at 32°C, 33.9±1 Gy. The time course of the development of the skin reactions was also very similar. Administration of TPPS (a hydrophilic photosensitiser) alone, prior to radiotherapy decreased this $ED_{50}$. Previous studies on the 'interaction' of radiotherapy and photosensitising drugs have produced conflicting results. Moan & Petterson (1981) failed to observe any modifying effects of haematoporphyrin or HPD (unlike TPPS, both lipophilic photosensitisers) on the sensitivity of NHIK 3025 cells to 220 kV X-rays under aerobic conditions. Similarly, Bellnier & Dougherty (1986) did not observe any change in the radiosensitivity of Chinese hamster ovary fibroblasts when they were pretreated with HPD. In contrast, however, i.p. injection of HPD 48 h prior to gamma-irradiation produced significant additional inhibition of tumour growth compared to radiotherapy alone in an *in vivo* clonogenic assay of a rat glioma model (Kostron *et al.*, 1986). When the effect of these treatments on the implanted tumours in the animals was assessed by an *in vitro* clonogenic assay, potentiation was again observed. In both assays the tumours were left on the animal for 5 days after treatment. Surprisingly, treatment by HPD alone, without ionising irradiation, produced inhibition in the *in vitro* clonogenic assay. This might have been due to the animals being housed in ambient light allowing a small photodynamic effect to occur and calls into question whether the observed potentiation of radiotherapy was truly independent of a photodynamic effect. Zhao *et al.* (1986) reported preliminary results from a clinical study on the use of HPD as a sensitiser for radiotherapy of oral and maxillofacial tumours. They claimed considerable enchancement of tumour destruction. The controls, however, were historical and had received a different radiotherapy schedule from the patients receiving HPD. From the above discussion, no clear picture emerges regarding the interaction of porphyrins 'alone' and radiotherapy. In our experimental protocol, it is possible but unlikely that the reduced $ED_{50}$ was due to a PDT effect caused by ambient lighting in the holding room. Light here was at low dose-rate (1 mW $cm^{-2}$, cf. 75 mW $cm^{-2}$ for the PDT exposures), with an absolute maximum daily dose of 7 J $cm^{-2}$ directly under the room lights (cf. the acute tolerance dose of 90 J $cm^{-2}$ for PDT). At no point were clinical signs such as erythema or oedema observed in these animals, which were retained for the overall duration of the longest experiments, nor was there any increase at any time in xenon $T_{\frac{1}{2}}$ (Moore, unpublished).

Intentional PDT prior to graded doses of radiotherapy produced a more marked decrease in the $ED_{50}$ than administration of drug alone. This reached significance at an interval between PDT and X-ray treatment of 5 days. Experiments reported previously (Benstead & Moore, 1988*b*) indicated that the level of TPPS would not be expected to change in mouse tail skin between 2 and 6 days after i.v. injection. Therefore this significant difference was unlikely to be due to an alteration in the level of the photosensitising drug. It suggests there was an interaction between the two modalities in the production of normal tissue damage, for which there are several possible explanations.

Both modalities produce chromosomal damage. Gomer (1980) observed DNA damage in the form of alkali-labile lesions and single strand breaks in Chinese hamster ovary cells treated with HPD phototherapy. Both X-rays and HPD plus light were found to induce chromosomal aberrations in NHIK cells (Evensen & Moan, 1982). If radiotherapy and PDT interacted by both inducing damage in DNA, rather than by a radiosensitising effect of the drug, then pretreatment with a tolerance dose of X-irradiation might be expected to increase the necrosis rate in mice treated with graded doses of PDT. This is consistent with the results observed here for TPPS (Figure 3). If the inhibition of repair of X-ray induced DNA strand breaks by PDT, reported by Boegheim *et al.* (1987) *in vitro* in murine fibroblasts, also occurs in the present experimental system, this would potentiate the interaction between the two modalities.

Alternatively, the increase in probability of necrosis with combined modality therapy might be due to interaction of the two modalities on the vascular system. Both produce changes in blood flow as inferred by alterations in xenon $T_{\frac{1}{2}}$. An increased blood flow on the first and seventh days after X-irradiation as evidenced by the significant shortening in xenon $T_{\frac{1}{2}}$ (Figure 2), might result in a rise in tissue oxygen level in the tails, which are usually hypoxic at room temperature (Hendry *et al.*, 1976). In view of the dependence of PDT on the presence of oxygen (Gomer & Razum, 1984), pre-treatment with ionising radiation could thus lead to sensitisation to subsequent PDT. Against this, however, is the observation that the decreased $ED_{50}$ persisted when the interval between X-rays and PDT was prolonged to 3 and 6 weeks, although the blood flow had returned to control levels.

If xenon $T_{\frac{1}{2}}$ is indeed predictive of impaired flow and reduced oxygenation, then the increased $T_{\frac{1}{2}}$ at 1 day after PDT might have been expected to lead to a *rise* in the $ED_{50}$ when the mice were treated at that time by X-rays, due to hypoxia of the target cells (comparable to the additional sparing of tail skin observed by Hendry (1978) on clamping tail skin prior to X-irradiation). Similarly, a fall in $ED_{50}$ might have been expected when the PDT interval was extended to 5 days, in view of the improvement in blood flow compared with control animals whose tails are, as we have noted, normally moderately hypoxic (Hendry, 1978). In contrast to these expectations, a 'tolerance' dose of PDT prior to X-irradiation produced a highly significant *decrease* in radiation $ED_{50}$ regardless of whether the interval between the two modalities was 1 or 5 days. The interaction between the two cannot therefore be explained on the basis of the altered blood *flow* after PDT.

Rubin and Casarett (1968) postulated that late radiation damage might be caused by leakage of plasma into the interstitial space as a result of compromise of the endothelial lining by irradiation. They suggested that this might stimulate fibrosis, thus comprising function. Increased vascular *permeability*, as revealed by the accumulation of intravenously administered radiolabelled albumin in tissues, has been demonstrated following both PDT (Lim *et al.*, 1985) and X-irradiation (Krishnan *et al.*, 1987). A histological study reported by us previously (Benstead & Moore, 1989) found that development of oedema exhibited different dose-response curves following PDT, indicating that this was probably not the main mechanism responsible for necrosis following PDT alone. It is possible, however, that the increase in interstitial fluid secondary to PDT might have an additive effect with that due to X-irradiation, thus increasing the probability of fibrosis and necrosis. This might provide an explanation for the prolonged period over which necrosis occurred following PDT in groups of mice which had been pretreated with ionising radiation.

A further possibility is that in tails pretreated with X-rays, the *angiogenic* response following PDT using TPPS reported previously (Benstead and Moore, 1989), by stimulating endothelial cells to divide, caused them to express potentially lethal damage induced by the X-rays. Endothelial cell death would prevent the recovery in blood flow normally observed following PDT, more endothelial cells might be stimulated to divide, causing yet more cell death, and so on in an avalanche effect. Reversing the combination, pretreatment by PDT 1 or 5 days prior to X-rays, might be expected to produce an increase in the proportion of dividing endothelial cells, which would express potentially lethal damage subsequent to irradiation, and hence reduce the $ED_{50}$ for damage to the dependent parenchyma.

In summary, combined modality treatment by PDT (with TPPS and full-spectrum light) and X-irradiation resulted in an increased incidence of necrosis of normal tissues. TPPS appeared to have a radiosensitising effect although we cannot yet definitively rule out a PDT effect mediated by the subdued ambient light under which the mice were housed. The effect of prolonging the interval between modalities on the necrosis rate, did not appear to be related to the time course of either the changes in blood flow (measured by xenon clearance) or the skin reactions following X-irradiation. Further experiments are underway to determine the precise mechanism(s) of interaction, but it is clear that dose-modificaton may be necessary if an unacceptable level of normal tissue injury is to be avoided following this combination of modalities.

### References

BELLNIER, D.A. & DOUGHERTY, T.J. (1986). Haematoporphyrin derivative photosensitisation and gamma irradiation damage interaction in Chinese hamster overy fibroblasts. *Int. J. Radiat. Biol.*, **50**, 659.

BENSTEAD, K. & MOORE, J.V. (1988*a*). Vascular function and the probability of skin necrosis after photodynamic therapy: an experimental study. *Br. J. Cancer*, **57**, 451.

BENSTEAD, K. & MOORE, J.V. (1988*b*). The effect of fractionation of light treatment on necrosis and vascular function of normal skin following photodynamic therapy. *Br. J. Cancer*, **58**, 301.

BENSTEAD, K. & MOORE, J.V. (1989). Quantitative histological changes in murine tail skin following photodynamic therapy. *Br. J. Cancer*, **59**, 503.

BOEGHEIM, J.P.P., DUBBELMAN, T.M.A.R., MULLENDERS, L.H.F. & VAN STEVENINCK, J. (1987). Photodynamic effects of haematoporphyrin derivative on DNA repair in murine L929 fibroblasts. *Biochem. J.*, **244**, 711.

CARRUTH, J.A.S. & MCKENZIE, A.L. (1985). Preliminary report of a pilot study of photoradiation therapy for the treatment of superficial malignancies of the skin, head and neck. *Eur. J. Surg. Oncol.*, **11**, 47.

DOUGHERTY, T.J. (1981). Photoradiation therapy for cutaneous and subcutaneous malignancies. *J. Invest. Dermatol.*, **77**, 122.

EVENSEN, J.F. & MOAN, J. (1982). Photodynamic action and chromosomal damage: a comparison of haematoporphyrin derivative (HpD) and light with X-irradiation. *Br. J. Cancer*, **45**, 456.

GILBERT, C.W. (1969). Computer programmes for fitting Puck and probit survival curves. *Int. J. Radiat. Biol.*, **16**, 323.

GOMER, C.J. (1980). DNA damage and repair in CHO cells following haematoporphyrin photoradiation. *Cancer Lett.*, **11**, 161.

GOMER, C.J. & RAZUM, N.J. (1984). Acute skin response in albino mice following porphyrin photosensitization under oxic and anoxic conditions. *Photochem. Photobiol.*, **40**, 435.

GRASCHEW, G. & SHOPOVA, M. (1986). Photodynamic therapy and gamma irradiation of tumours: effect of tumour-cell reoxygenation. *Lasers Med Sci.*, **1**, 193.

HENDRY, J.H., ROSENBERG, I., GREENE, D. & STEWART, J.G. (1976). Tolerance of rodent tails to necrosis after daily fractionated X-rays or D-T neutrons. *Br. J. Radiol.*, **49**, 690.

HENDRY, J.H. (1978). Radionecrosis of normal tissue: studies on mouse tails. *Int. J. Radiat. Biol.*, **33**, 47.

HENDRY, J.H. (1980). Analysis of the steepness of the dose-incidence curve in mouse tails after a multifraction X-ray schedule. *Radiology*, **134**, 757.

HENDRY, J.H., RUSHTON, D.A. & ALLEN, T.D. (1982). Epidermal kinetics and ultrastructure of tolerance to radionecrosis in mouse tails. *Radiat. Res.*, **89**, 513.

HUGH-JONES, P. & GARDNER, W.N. (1987). Laser photodynamic therapy for inoperable bronchogenic squamous carcinoma. *Q. J. Med.*, **64**, 565.

KAYE, A.H., MORSTYN, G. & BROWNBILL, D. (1987). Adjuvant high dose photoradiation therapy in the treatment of cerebral glioma: a phase 1-2 study. *J. Neurosurg.*, **67**, 500.

KETY, S.S. (1949). Measurement of regional circulation by the local clearance of radioactive sodium. *Am. Heart J.*, **38**, 321.

KOSTRON, H., SWARTZ, M.R., MILLER, D.C. & MARTUZA, R.L. (1986). The interaction of hematoporphyrin derivative, light and ionizing radiation in a rat glioma model. *Cancer*, **57**, 964.

KRISHNAN, E.C., KRISHNAN, L., JEWELL, B., BHATIA, P. & JEWELL, W.R. (1987). Dose dependent radiation effects on microvasculature and repair. *J. Natl Cancer Inst.*, **79**, 1321.

LAM, S., KOSTASHUK, E.C., COY, E.P. & 4 others (1987). A randomized comparative study of the safety and efficacy of photodynamic therapy using Photofrin II combined with palliative radiotherapy versus palliative radiotherapy alone in patients with inoperative obstructive non-small cell bronchogenic carcinoma. *Photochem. Photobiol.*, **46**, 893.

LIM, H.W., YOUNG, L., HAGAN, M. & GIGLI, I. (1985). Delayed phase of haematoporphyrin induced phototoxicity: modulation by complement, leukocytes and antihistamines. *J. Invest. Dermatol.*, **84**, 114.

MOAN, J. & PETTERSON, O. (1981). X-irradiation of human cells in culture in the presence of haematoporphyrin. *Int. J. Radiat. Biol.*, **40**, 107.

NSEYO, U.O., DOUGHERTY, T.J. & SULLIVAN, L. (1987). Photodynamic therapy in the management of resistant lower urinary tract carcinoma. *Cancer*, **60**, 3113.

OHNISHI, Y., YAMANA, Y. & MINEI, M. (1986). Photoradiation therapy using argon laser and a haematoporphyrin derivative for retinoblastoma – a preliminary report. *Jap. J. Ophthalmol.*, **30**, 409.

RUBIN, P. & CASARETT, G.W. (1968). *Clinical Radiation Pathology, Vol. 1*, p. 46. W.B. Saunders: New York.

SCHUH, M., NSEYO, U.O., POTTER, W.R., DAO, T.L. & DOUGHERTY, T.J. (1987). Photodynamic therapy for palliation of locally recurrent breast carcinoma. *J. Clin. Oncol.*, **5**, 1766.

SIEGEL, S. (1956). *Nonparametric Statistics for Behavioral Science.* McGraw-Hill: New York.

THOMAS, R.J., ABBOTT, M., BHATHAL, P.S., ST JOHN, D.J.B. & MORSTYN, G. (1987). High dose photoradiation of oesophageal cancer. *Ann. Surg.*, **206**, 193.

WARD, B., FORBES, I.J., COWLED, P.A., MCEVOY, M.M & COX, L.W. (1982). The treatment of vaginal recurrences of gynaecologic malignancy with phototherapy following haematoporphyrin derivative pretreatment. *Am. J. Obstet. Gynecol.*, **142**, 356.

WINTHER, J., OVERGAARD, J. & EHLERS, N. (1988). The effect of photodynamic therapy alone or in combination with misonidazole or X-rays for management of a retinoblastoma-like tumour. *Photochem. Photobiol.*, **47**, 419.

ZHAO, F.Y., ZHANG, J.H., HUANG, H.N., SUN, K.L., LING, Q.B. & XU, B. (1986). Use of haematoporphyrin derivative as a sensitizer for radiotherapy of oral and maxillofacial tumours: A preliminary report. *Lasers Med. Sci.*, **1**, 253.